# Author's overview: identifying SARS-CoV-2 antiviral compounds

**DOI:** 10.1042/BCJ20210426

**Published:** 2021-07-02

**Authors:** John F.X. Diffley

**Affiliations:** Chromosome Replication Laboratory, The Francis Crick Institute, 1 Midland Road, London NW1 1AT, U.K.

**Keywords:** antiviral, coronavirus, COVID-19

## Abstract

In response to the COVID-19 pandemic, we began a project in March 2020 to identify small molecule inhibitors of SARS-CoV-2 enzymes from a library of chemical compounds containing many established pharmaceuticals. Our hope was that inhibitors we found might slow the replication of the SARS-CoV-2 virus in cells and ultimately be useful in the treatment of COVID-19. The seven accompanying manuscripts describe the results of these chemical screens. This overview summarises the main highlights from these screens and discusses the implications of our results and how our results might be exploited in future.

Coronaviruses encode multiple enzymes important for viral replication [1], and any of these could be attractive drug targets. We identified inhibitors of seven enzymes, described in the accompanying papers. Table 1 summarises the viral enzymes that control SARS-CoV-2 replication, and the best hits obtained for each that we screened.

**Table 1:** Summary of the best hits from each of the seven screens

Enzyme	Inhibitor	Description	Enzymatic inhibition (IC_50_)	Inhibition of virus growth (EC_50_)
Nsp3 papain-like protease	Dihydrotanshinone I	a naturally occurring compound extracted from Salvia miltiorrhiza	0.5 µM	8 µM
Nsp3 de-mono ADP ribosylase	Not screened	n/a	n/a	n/a
Nsp5 main protease	Calpain inhibitor I	synthetic tripeptide (ALLN) aldehyde; potent inhibitor of cysteine proteases	2.0 µM	0.3 µM
	Z-AVLD-FMK	FMK derivative based on nsp5 cleavage sequence	<1 nM	65 µM
Nsp12/7/8 RNA-dependent RNA polymerase (RdRp)	GSK-650394	serum and glucocorticoid-activated kinase-1 and 2 inhibitor.	31 µM	7.6 µM
	C646	Histone acetyltransferase (p300) inhibitor	6 µM	19 µM^1^
	Suramin (also NF 023, PPNDS, Evans Blue, Diphenyl Blue)	Used to treat African sleeping sickness and river blindness.	0.5 µM	10 µM
Nsp13 RNA helicase	FPA-124	Akt inhibitor. Induces apoptosis.	9 µM	14 µM^1^
	Suramin (also NF 023, PPNDS, Evans Blue, Diphenyl Blue)	Used to treat African sleeping sickness and river blindness.	1 µM	10 µM
Nsp14 mRNA G-N7 cap methyltransferase	PF-03882845	Non-steroidal mineralocorticoid antagonist. Orally bioavailable.	1 µM	11 µM
	Trifluperidol	Anti-psychotic butyrophenone	13 µM	15 µM
	Inauhzin	Cell permeable SIRT1 deacetylase inhibitor.	23 µM	13 µM
	Lomeguatrib	O^6^-methylguanine-DNA-methyltransferase (MGMT) inhibitor used in combination with temozolomide in melanoma treatment. Well tolerated alone.	60 µM	60 µM
Nsp14/10 exoribonuclease	Patulin	Mycotoxin produced by Aspergillus and Penicillium	2 µM	5 µM^1^
	Aurintricarboxylic acid	Inhibits wide range of enzymes in nucleic acid metabolism. Previously shown to inhibit SARS replication.	10 µM	10 µM
Nsp15 endoribonuclease	NSC 95397	Cdc25 phosphatase inhibitor	43 µM	none
Nsp16/10 ribose 2′-O-cap methyltransferase	Not screened	n/a	n/a	n/a

1 Cytotoxic compounds.

Our screens identified several drugs that might be repurposed to treat COVID-19. Suramin, which we identified as inhibiting both nsp13 RNA helicase and nsp12/7/8 RdRp, is used to treat African sleeping sickness and river blindness [2,3]. Interestingly, suramin also affects the growth of a wide range of viruses, in part by interfering with virus-receptor interactions [2,4–6]. Two of the nsp14 cap methyltransferase inhibitors are also clinically relevant. Trifluperidol is used in the treatment of mania and schizophrenia. Lomeguatrib is an inhibitor of O^6^-methylguanine-DNA-methyltransferase [7] and has been used to enhance the effects of DNA alkylating agents including Temozolomide. A better understanding of the side effects associated with the doses of these drugs required to suppress viral growth is needed before considering them for clinical use.

Other inhibitors are further from the clinic. PF-03882845, another nsp14 cap methyltransferase inhibitor, is a non-steroidal mineralocorticoid antagonist [8] which has been shown to be orally bioavailable and well-tolerated in humans. GSK-650394, which we found as an RdRp inhibitor, is an inhibitor of serum and glucocorticoid-activated kinase (SGK1) [9]. Interestingly, GSK-650394 has previously been shown to inhibit Influenza virus replication in cell models [10]. Dihydrotanshinone I was the best nsp3/PLpro inhibitor we found and also inhibited viral growth *in vitro*. Dihydrotanshinone I is a naturally occurring compound from the plant *Salvia miltiorrhiza* and did not exhibit cytotoxicity in our experiments. Further tests of efficacy and toxicity in animal models might determine whether dihydrotanshinone I has potential in the clinic.

Our screens also identified compounds that might be leads for further drug development. For example, by optimising the peptide sequence of a peptidomimetic Caspase inhibitor we found in the screen, we were able to generate an inhibitor of nsp5 Main protease with an IC_50_ of less than 1 nanomolar. Though this drug (Z-AVLD-FMK) was relatively ineffective in preventing virus replication in cell culture, it represents a good starting point for developing better, perhaps more bioavailable, inhibitors. It may ultimately be important to obtain atomic resolution structures of each of the enzymes with their inhibitors to inform the chemistry required to improve inhibitor efficacy.

Our inhibitors, taken together, comprise a nascent ‘chemical toolbox’ for studying SARS-CoV-2. Such a toolbox could be used to study the roles of these enzymes in the intracellular phase of the coronavirus cycle, identifying major vulnerabilities. Moreover, by determining the effects of combinations of inhibitors on viral growth, novel approaches to combination therapies may be identified. In this regard, the efficacy of the nsp14 methyl transferase inhibitors was enhanced by combination with remdesivir, suggesting an interesting possibility for a combination therapy. Nsp14/10 has been hypothesised to act as a ‘proofreading exonuclease’ during virus replication [11–13]. Interestingly, nsp14/10 exonuclease inhibitors did not exhibit any additive effects with remdesivir. Remdesivir acts as a delayed chain terminator, terminating three nucleotides after its incorporation [14,15], and thus may evade proofreading by nsp14/10. It will be interesting to examine this possibility with the purified proteins and to determine if nsp14/10 inhibitors make direct chain terminators more effective in inhibiting viral replication. Many of the enzymes we examined are highly conserved in coronaviruses. It will be important to test these inhibitors across a wide range of coronaviruses.

By interfering with the intracellular phase of the virus life cycle, these inhibitors may cause accumulation of partially replicated, or partially capped cytoplasmic RNAs. Such structures are known to elicit cellular antiviral responses, so the inhibitors we have identified may, in addition to directly inhibiting viral replication, also facilitate cellular responses to viral infection. Further work is needed to investigate this possibility.

There are many other classes of viruses that have the potential to cause serious health problems in the future. Structural viral coat proteins tend to evolve rapidly, making it difficult to develop vaccines before new threats emerge; however the enzymology involved in viral replication evolves more slowly, suggesting that inhibitors with broad efficacy across viral groups could be developed and would be useful as frontline defence while vaccines are developed. We hope that this project will help convince governments to coordinate efforts to develop antivirals to virus groups before they become problems.
